# Nanomaterials for Cortisol Sensing

**DOI:** 10.3390/nano12213790

**Published:** 2022-10-27

**Authors:** Giuseppe Trusso Sfrazzetto, Rossella Santonocito

**Affiliations:** 1 Department of Chemical Sciences, University of Catania, Viale A. Doria 6, 95100 Catania, Italy; 2 National Interuniversity Consortium for Materials Sciences and Technology (I.N.S.T.M.), Research Unit of Catania, 95100 Catania, Italy

**Keywords:** sensing, nanomaterials, cortisol, point of care

## Abstract

Space represents one of the most dangerous environments for humans, which can be affected by high stress levels. This can lead to severe physiological problems, such as headaches, gastrointestinal disorders, anxiety, hypertension, depression, and coronary heart diseases. During a stress condition, the human body produces specific hormones, such as dopamine, adrenaline, noradrenaline, and cortisol. In particular, the control of cortisol levels can be related to the stress level of an astronaut, particularly during a long-term space mission. The common analytical methods (HPLC, GC-MS) cannot be used in an extreme environment, such as a space station, due to the steric hindrance of the instruments and the absence of gravity. For these reasons, the development of smart sensing devices with a *facile* and fast analytical protocol can be extremely useful for space applications. This review summarizes the recent (from 2011) miniaturized sensoristic devices based on nanomaterials (gold and carbon nanoparticles, nanotubes, nanowires, nano-electrodes), which allow rapid and real-time analyses of cortisol levels in biological samples (such as saliva, urine, sweat, and plasma), to monitor the health conditions of humans under extreme stress conditions.

## 1. Introduction

The extraterrestrial environment, especially during long-term space missions, is one of the most hostile places for humans and leads to high levels of stress in astronauts. In particular, persistent chronic stress can lead to major physiological problems, such as headaches, insomnia, fatigue, hypertension, loss of concentration, gastric and memory problems, depression, and immune system dysfunctions [1,2,3,4,5,6,7]. To avoid having a chronic response to stress, during a space mission, whether short or, especially, in the long term, it is essential to monitor daily stress levels to avoid compromising astronauts’ activities. During a stress condition, the human body synthesizes catecholamine neurotransmitters (CNs) such as dopamine (DA), adrenaline (AD), and noradrenaline (NAD), and also specific hormones (called ‘stress hormones) [8], the most important of which is cortisol. Cortisol is a fat-soluble steroid hormone, one of the main glucocorticoids synthesized by the adrenal cortex [9]. It plays a key role in certain physiological processes such as the regulation of blood pressure, glucose levels, and carbohydrate metabolism. Cortisol is a stress biomarker, and its release is regulated by the hypothalamic-pituitary-adrenal (HPA) axis response [10], which is the central stress response system in humans. The HPA axis is characterized by the hypothalamic release of corticotropin-releasing factor (CRF).

**Figure 1:**
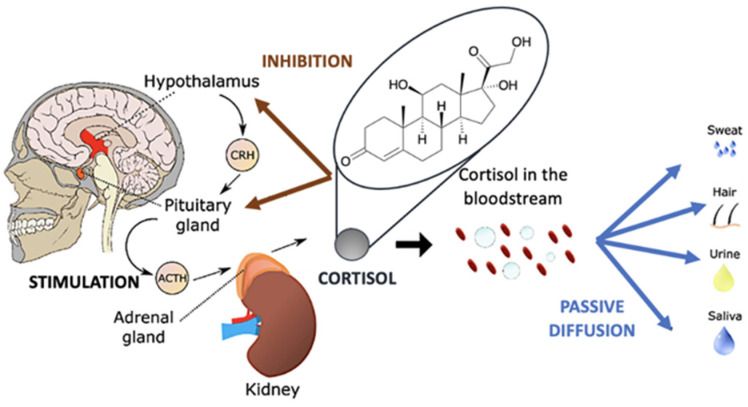
Mechanism of cortisol generation in the human body. Free cortisol is released into the bloodstream and diffused to different tissues (adapted with permission from [17]. Copyright 2020 Elsevier).

Figure 1 shows schematically the mechanism of the release of adrenal cortisol into the blood and diffusion into other tissues. In particular, CRF binds to its receptors in the anterior pituitary gland, releasing adrenocorticotropic hormone (ACTH), which then binds to receptors in the adrenal cortex, stimulating cortisol release [11]. The cortisol secreted will then participate in all ongoing physiological processes. For these reasons, monitoring cortisol levels in real time should be very useful during a space mission to control stress levels.

Cortisol levels change during the day following a well-established circadian rhythm, they are lowest around midnight and start to increase in the early morning, and decrease again during the day [12,13,14]. Cortisol can be found in detectable amounts in different biological fluids such as blood, urine, saliva, sweat, hair, and interstitial fluid (ISF), as reported in Table 1. In particular, blood is the matrix in which the highest concentration of cortisol is present; however, its sampling is not recommended because blood collection requires specialized medical personnel and sterile equipment, with the constant risk of infection [15]. High concentrations of cortisol can be found in *urine*, and the measurement of these levels requires a sampling collection over 24 h. Although it is not an invasive method, it has several disadvantages in terms of practicality and reliability, because in a space mission, all urine is collected, concentrated, filtered, and purified to obtain drinkable water. Even if containing a lower concentration of cortisol than blood, *saliva* is the preferred body fluid for the detection of cortisol, as sampling for analysis is almost entirely non-invasive, with little or no discomfort for the subject providing the sample. Furthermore, the ease of sample collection, handling, and storage has increased the prospects of its application in *point-of-care* sensors for real-time cortisol detection [16].

Cortisol can also be found in *sweat* and *hair*, although at low concentrations, and it is hypothesized that there is a strong correlation between the cortisol levels in sweat and hair due to the pathway cortisol takes from serum, sebum, and sweat to hair. The sampling of cortisol in sweat is inconvenient as sweating depends on atmospheric conditions (humidity and temperature) and physical activity levels. However, research is developing sweat collection systems (e.g., a microfluidic cloth or patch) but these have not yet been tested in space environments [18]. ISF is an extracellular fluid that surrounds the cells of the human body. Its composition is similar to that of blood plasma. Cortisol, along with other metabolites and proteins, can be found within the ISF, but the detection of the hormone may require an invasive approach [19]. Taking into account these considerations, the preferred human matrixes to detect cortisol levels are saliva and sweat.

Enzyme-linked immunosorbent assays (ELISA) [20], chemiluminescence assays (CLIAr) [21], and capillary electrophoresis-based immunosorbent assays (CE-IA) [22] are the common techniques used to quantify free cortisol levels in human patients. In addition, immunosensing or immunoassay (IA) techniques are very promising for screening analyses as they improve sensitivity [23,24], with a low limit of detection (LOD) values, and are cheap and easy to use. The common laboratory analytical methods, such as LC-MS [25], HPLC, and, in particular, tandem mass spectrometry (MS/MS) are also extremely sensitive [26], accurate, and selective, but cannot be used in ‘extreme’ environments such as space stations or spacecraft due to their bulkiness. In addition, they need trained personnel, and the lack of gravity (or reduced gravity) affects their operation. Therefore, due to the change in cortisol levels depending on daily activities, the development of *point-of-care* (POC) sensing devices that qualitatively and quantitatively monitor cortisol levels through a fast and easy analytical protocol can be extremely useful for space applications.

The target of NASA and ESA for the future is to start long space missions, in order to create permanent space bases on the moon and to reach Mars. These future missions preclude the possibility to collect a sample on the mission and perform the analysis on Earth which leads to the necessity to execute the analysis in situ. Recently, tissue- and clothing-based biosensing approaches are growing [27], allowing diagnostic devices to be integrated into a wearable format, which can be used to develop non-invasive approaches for the quantitative measurement of biomarkers in bodily fluids. In addition, many methods have been employed to improve the use of Lateral Flow Assays (LFAs) [28], one-step assays that require low sample volumes to produce good qualitative, quantitative, or semi-quantitative results. There are LFAs for serum, salivary, plasma, and sweat cortisol that provide non-invasive techniques. Now, research wants to develop multiplexed LFAs for cortisol as they could improve the application of these devices, allowing a POC differential diagnosis of the disease. In this context, smartphone applications have been developed for LFAs for cortisol, LH (Luteinizing hormone), and TSH (Thyroid-stimulating hormone), allowing patients to monitor their health at home [29].

This review aims to summarize recent (from 2011) miniaturized sensoristic devices based on nanomaterials (gold and carbon nanoparticles, nanotubes, nanowires, nano-electrodes), which allow rapid and real-time analyses of cortisol levels in biological samples (such as saliva, urine, sweat, and plasma), to monitor the health conditions of humans under extreme stress conditions. In particular, we focused our attention on optical and electrochemical sensing of cortisol by nanostructures, due to their high sensitivity.

Recently, some interesting reviews on the electrochemical [17], immunological [30], and aptameric sensing of cortisol with point-of-care technologies have been reported [31].

## 2. Nanomaterials for Sensing

In recent years, nanomaterials have been widely used for the chemical sensing of many species [32,33,34,35]. In particular, cortisol sensing by nanostructured species can be performed by different classes of nanosystems: gold nanoparticles, carbon nanoparticles, graphene oxide, metal nanoflowers, nanowires, nanoflakes, and metal nanorods. Gold nanoparticles (AuNPs) have an average diameter of about 16–50 nm, excellent physical and chemical properties, good biocompatibility, and very high molar absorbance, which makes them excellent luminescence quenchers employed in sensing applications [36]. Carbon-based fluorescent nanoparticles (CNPs) [37,38,39], such as carbon dots (CD), graphene quantum dots (GQD), and polymer dots (PD), have a narrow size distribution ranging from about 3.0 to 6.0 nm and an average diameter of about 5.1 nm. Graphene oxide is a 2D nanostructure based on SP2-carbon atoms, which contains different oxygen atoms due to the carbon oxidation reaction [40]. Metal nanoflowers are composed of metal phosphates such as cobalt [41], copper, manganese, zinc, ferrous, and calcium salts as inorganic components, having larger diameters concerning carbon and gold nanoparticles (100–500 nm). They are characterized by simple synthesis, high stability, larger specific area, good carrier transmission, and enhanced photocatalytic efficiency. Nanowires show high electrical conductivity, good optical properties, high flexibility, and a low cost of synthesis [42]. For these reasons, they have been used in the realization of various transparent, flexible, and stretchable devices, such as touch screens, smart windows, and solar cells. Nanoflakes are in the range of 1.5–5 nm in diameter and contain different oxygen-based groups, such as epoxide, ether, and hydroxyl groups, and a wide type of morphologies (hexagonal, triangular, rectangular). Nanorods can be obtained from different metal or non-metal elements and show interesting chemical–physical properties [43]. Their dimensions are wide, from 1 to 100 nm, and are used in many electronic devices.

The use of these nanostructures in sensing applications drastically improves the selectivity, sensitivity, and stability of the sensor, due to the improvements of nanomaterials in (*i*) catalytic activity; (*ii*) the conductivity of the transducer due to its excellent mechanical, thermal, electrical, and optical properties; (*iii*) several receptors that can be anchored onto the nanostructure; (*iv*) high surface-to-volume ratio; (*v*) material sizes that can fall into the nanometre region, drastically increasing the number of surface atoms. Furthermore, the surface of nanostructures can be functionalized with pendant arms leading to increased reactivity [44].

In addition, nanostructures improve the affinity of the analyte-receptor bond since there is a significant delocalization of the electrical charge on the surface [45]. Furthermore, there are several methods to detect cortisol levels that exploit both optical and electrical output.

## 3. Optical Nanosensors

**Figure 2:**
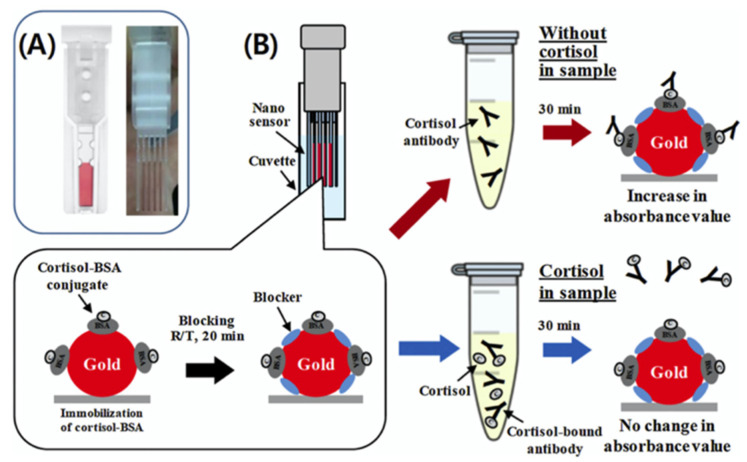
(**A**) Real image of a Plex NanoCuve before (right) and after (left) installation in a cuvette. (**B**) Schematic of the competitive cortisol test with the Plex NanoCuve (reproduced with permission from [46] Copyright 2018 Elsevier).

Jeon and co-workers have designed a new localized surface plasmon resonance-based cuvette-type sensor for the detection of cortisol, leading to rapid detection in human serum samples [46]. In particular, the nanosensor, called Plex NanoCuve, consists of a parallel assembly of four sensing strips, two protection strips, and two spacer strips. The principle of operation is based on the change in the local refractive index of gold nanoparticles upon binding to biomolecules, this change induces a shift in the localized surface plasmonic resonance (LSPR) wavelength [47,48]. In this nanosensor, conjugated cortisol binds bovine serum albumin (BSA) and is immobilized on plastic units coated with gold nanoparticles (Figure 2). Cortisol was detected in PBS solution and serum (within 20 min) at concentrations between 1 and 10,000 ng/mL, values comparable to that of the traditional enzyme-linked immunosorbent assay (ELISA), which typically requires more than 4 h and complex sample preparation. The few amounts of samples (200 μL) required for a single analysis and the relatively quick time for the analysis should be fine for space applications after reducing or changing the readout system and obtaining a portable device.

**Figure 3:**
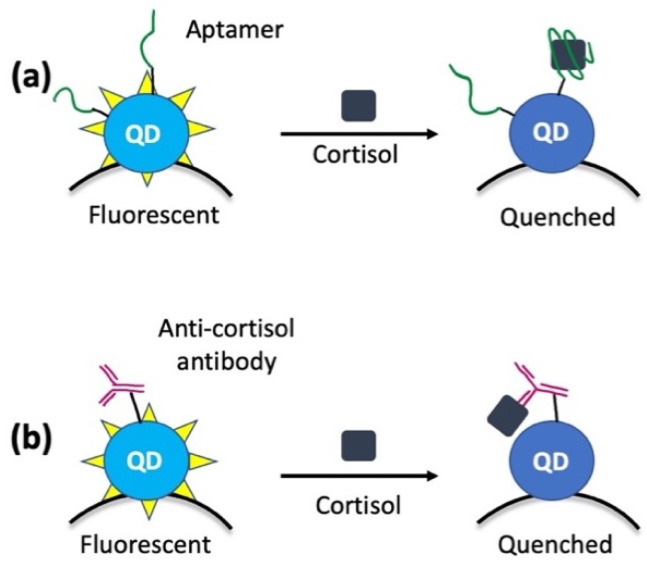
Target-induced quenching for cortisol detection; efficiency is modulated by the amount of cortisol captured on each quantum dot: (**a**) quantum dots conjugated with aptamers and (**b**) quantum dots conjugated with antibodies and transported by a magnetic nanoparticle (MNP).

Liu and co-workers developed a new type of portable biosensor based on carbon quantum dots (QDs) conjugated with magnetic nanoparticles (MNPs) for the sensitive detection of cortisol in saliva samples [49]. Firstly, they demonstrated that cortisol leads to the fluorescence quenching of QDs [50], functionalized on the surface with selective aptamers (Aptamer-QD@MNP) or antibodies (Antibody-QD@MNP), which are selective for cortisol (Figure 3). Then, they obtained the real nanosensor for the detection of cortisol in saliva samples by the conjugation between QDs and MNPs [51]. This approach allows the detection of cortisol concentrations lower than 1 nM. In particular, the detection limit obtained is ca. 1 nM for Aptamer-QD@MNP and ca. 100 pM for Antibody-QD@MNP.

**Figure 4:**
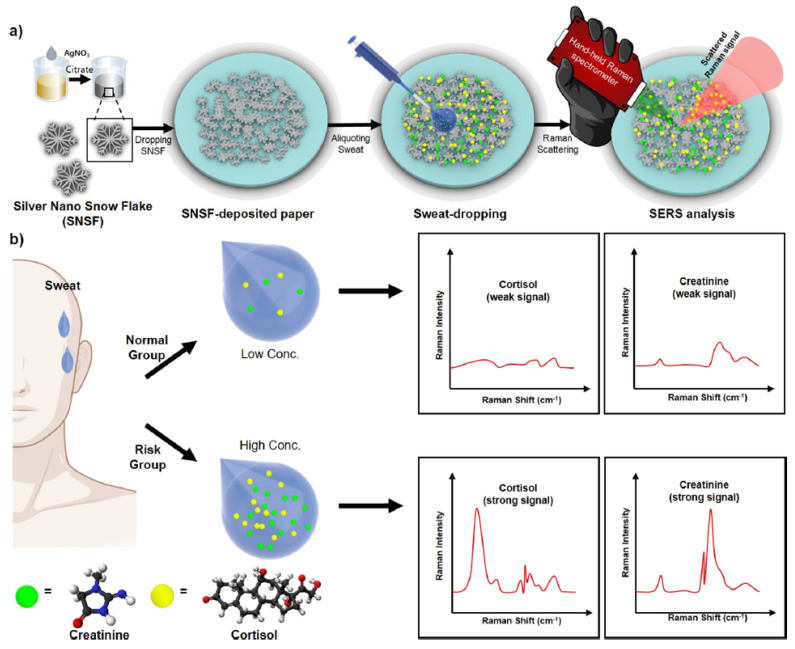
(**a**) Detection of stress biomarkers in sweat, (**b**) sweat sampling for the classification of normal and risk groups (reproduced with permission from [52]. Copyright 2021 American Chemical Society).

Kim and co-workers presented a new sensor to simultaneously detect creatinine (an important bioindicator of kidney function) and cortisol in sweat using a portable Raman spectrometer [52] (Figure 4). They fabricated silver nano snowflakes (SNSFs) on a SERS (surface-enhanced Raman scattering) substrate and hydrophobic filter paper using the polyol method [53]. In particular, the sensor was created by synthesizing a colloidal suspension of SNSF according to the polyol method reported in the literature [54]. This substrate facilitates the simultaneous detection of creatinine and cortisol and allows measurement on small sample volumes of human sweat (2 μL). Notably, the possibility to use a hand-held Raman spectrometer for these measurements leads to real applicability in a space mission.

**Figure 5:**
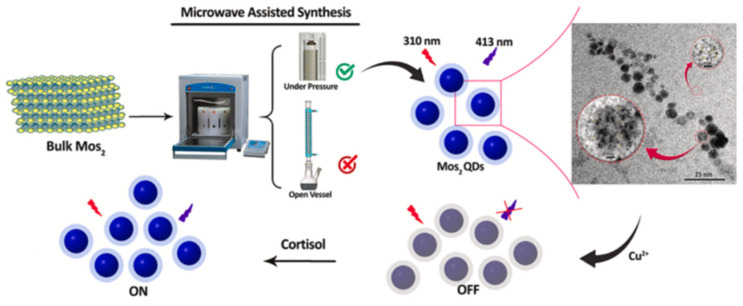
Overview of the microwave preparation of the on–off sensor for cortisol (adapted with permission from [55]. Copyright 2022 Elsevier).

Mohammad-Andashti and co-workers prepared an on-off fluorescent sensor for the detection of cortisol [55], based on highly luminescent MoS_2_ quantum dots (QDs) obtained by simple, environmentally friendly, and green microwave exfoliation (Figure 5). Specifically, high-fluorescence MoS_2_ quantum dots were prepared by mixing MoS_2_ powder with ammonium hydroxide and deionized water, transferred to a Teflon microwave vessel, hermetically sealed, and placed in a microwave oven. The detection of cortisol used an *on–off* protocol, in that some cations (such as Cu^2+^, Pb^2+^, Cd^2+^, Ag^+^, Fe^3+,^ and Al^3+^) switched off the luminescence intensity of MoS_2_ QDs, but then the luminescence of MoS_2_ QDs-Ag^+^ and Cu^2+^ mixtures was recovered with the addition of cortisol, with a higher emission recovery for the Cu^2+^ ion. This occurs because cortisol interacts with copper ions through its hydroxyl and carbonyl groups leading to a copper-cortisol complex, which releases copper from the surface of MoS_2_, resulting in the recovery of the emission intensity of MoS_2_ QDs.

The method was successful in determining the cortisol content in saliva, with a detection limit between 100 ng/mL and 500 ng/mL. Considering that the low level of cortisol in the saliva is from 1 to 11 ng/mL, the amount of sample required for a single analysis with this method must be 5 mL, so its application in real life is actually precluded.

Yılmaz and co-workers developed a plasmonic sensor based on gold nanoparticles for the real-time determination of cortisol in complex aqueous solutions [56]. The sensor surfaces were modified with propyl 3-(trimethoxyl)methacrylate, and then a precomplex was prepared using the functional monomer *N*-methacryloyl-L-histidine methyl ester, and then 2-hydroxyethyl methacrylate was added to the monomer solution to ethylene glycol dimethacrylate, and polymerization was initiated. Two plasmonic sensors, one imprinted with cortisol without AuNPs and one unimprinted without cortisol, were prepared to confirm the signal-enhancing effect of AuNPs and to determine the selectivity of the imprinting process, respectively. Finally, through the calculation of kinetic binding parameters, the detection performance of cortisol was evaluated, in particular showing a detection limit of 0.0087 ppb. The main limitation of this system is its non-portability, which precludes its use for real-time cortisol analysis. This problem can be solved by using a portable read-out system, such as optical fiber or a simple camera.

**Figure 6:**
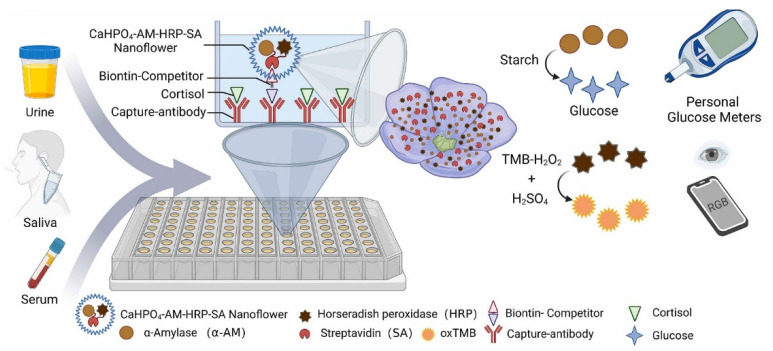
Schematic representation of cortisol sensing by dual-mode platform (reproduced with permission from [57]. Copyright 2022 Elsevier).

Very recently, Chen and co-workers developed a nanosensor based on calcium nanoflowers mixed with horseradish peroxidase, streptavidin, and α-amylase for cortisol detection, with a limit of detection of 95.5 pg/mL and response in the range of 0.33–1000 ng/mL [57]. They used this new sensor in rat serum, human urine, and saliva. In particular, this system exploits two different output signals, the change of glucose (by simple glucose blood meter) and TMB (Tetramethylbenzidine) oxidation (by a simple smartphone). Notably, this nanosensor can reveal the antidepressant effects of many drugs (Figure 6). The easy use of this prototype and the non-invasive detection method pave the way for the practical use of this system for cortisol detection.

## 4. Electrochemical Nanosensors

**Figure 7:**
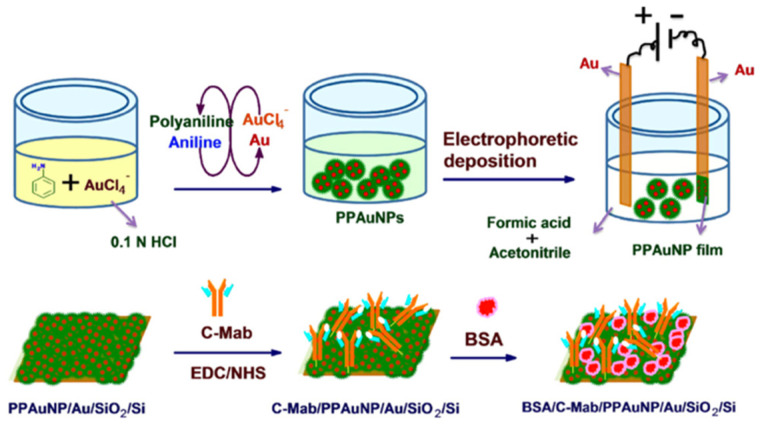
Schematic illustration of the fabrication of the BSA/C-Mab/PPAuNP/Au electrode for cortisol detection (reproduced with permission from [62]. Copyright 2015 Elsevier).

Recently, new electrochemical techniques for the detection of cortisol have gained popularity [58]. These techniques show a low cost, high sensitivity, and a high level of integration, especially since they can be portable [59,60,61]. In this context, Arya and co-workers designed an electrochemical biosensor for cortisol detection [62]. They electrophoretically deposited polyaniline-protected gold nanoparticles (PPAuNPSs) onto a gold electrode, and the cortisol-specific monoclonal antibody (C-M_ab_) was covalently immobilized on the electrode surface (PPAuNP/Au) using N-ethyl-N′-(3-dimethyl aminopropyl) carbodiimide and N-hydroxysuccinimide (EDC/NHS) as coupling agents (Figure 7). Using Cyclic Voltammetry (CV) and Differential Pulse Voltammetry (DPV), the concentration of cortisol in phosphate-buffered saline (PBS) solution was determined. The results showed that the PPAuNP-based electrode remained stable during repeated scans and, in addition, the BSA/C-Mab/PPAuNP/Au electrode in PBS buffer accurately detected cortisol in the range of 1 pM-100 nM, with a sensitivity of 1.63 μA/M. This system is optimized for the laboratory scale and requires implementation to obtain a point-of-care device.

**Figure 8:**
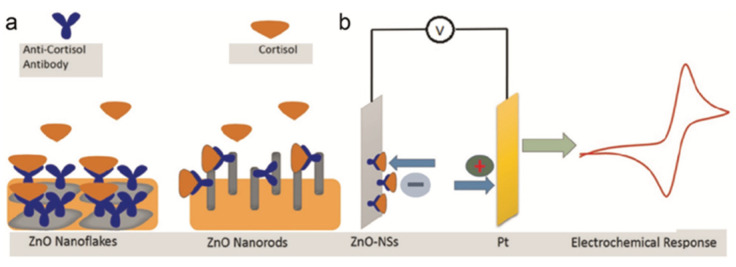
(**a**) The fabrication of the electrochemical immunosensor for the detection of cortisol based on ZnO-NRs and ZnO-NFs, prepared by the sonochemical method and the immobilization of the anti-cortisol monoclonal antibody on ZnO-NSs (**b**) Response obtained by the CV of the fabricated electrodes (reproduced with permission from [63]. Copyright 2015 Elsevier).

Vabbina and co-workers presented a highly sensitive and selective label-free electrochemical immunosensor for cortisol [63]. These sensors are one-dimensional ZnO nanorods (1D ZnO-NRs) and two-dimensional ZnO nanoflakes (2D ZnO-NFs), synthesized on Au-coated substrates in a one-pot approach. One-dimensional ZnO-NRs and two-dimensional ZnO-NFs were chosen because they offer detection advantages over bulk materials [64], in particular, the 1D-NSs have a high surface area/volume ratio, and the 2D-NSs have a large area in the polarized plane (0001) and a high surface charge density, such that they promote a higher loading of anti-cortisol antibodies (Anti-C_ab_) and thus improve detection performance. This is because the selective detection of cortisol by CV was achieved by immobilizing the anti-cortisol antibody (Anti-Cab) on ZnO nanostructures (NSs) via electrostatic interaction. These immunosensors, through CV studies on electrochemical detection, showed a high sensitivity of 11.86 mA/M and a minimum detection concentration of 1 pM. For real applications, the developed immunosensor was tested on human salivary cortisol of two specimens collected at different time intervals (see Figure 8). In addition, the shelf-life of this system is ca. 30 days, thus supporting its usability as a real detection system.

**Figure 9:**
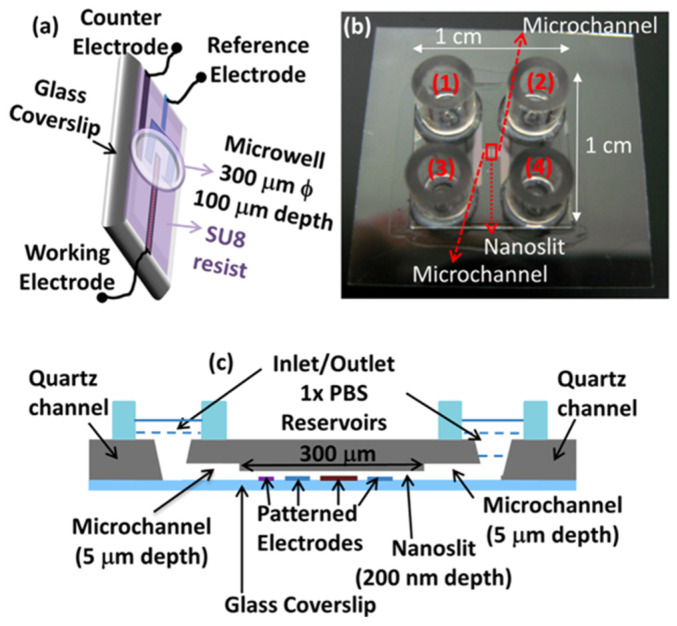
(**a**) Schematic view of the microwell device; (**b**) top view of the nanoslit device; and (**c**) schematic cross-sectional view of the nanoslit geometry (reproduced with permission from [65] Copyright 2015 Elsevier).

Sanghavi and co-workers presented a microfluidic aptamer-based quantitative cortisol detection methodology that does not require the labeling of the target, the immobilization of the capture probe on the detection surface, or washing steps before reading [65]. They used gold nanoparticles functionalized with aptamers (AuNPs) at high concentrations to increase the surface available for the capture of the analyte and to allow free three-dimensional diffusion of the analyte towards the binding surface. The detection is performed on an alternative graphene-modified electrode (Figure 9), optimized to enhance the adsorption and electron transfer kinetics of electroactive molecules. To improve the binding kinetics of aptamers for cortisol, the authors applied this sensing strategy without surface immobilization within a nanoslit (Figure 9) geometry, locating the sensing electrode in the vicinity of the electroactive species released by the aptamers. The microfluidic nature of this system precludes the possibility to use it in the absence of gravity, but it is open to the practicability for analysis in real life [66]. In fact, fluid motion and handling microgravity or the absence of gravity require different system engineering with respect to those used on Earth. In particular, in normal gravity conditions, buoyancy plays a crucial role in transport phenomena, while in the absence of gravity, the main effect involved in fluid transport is surface tension. For this reason, microfluidic systems developed on Earth must be implemented to be used in space missions.

**Figure 10:**
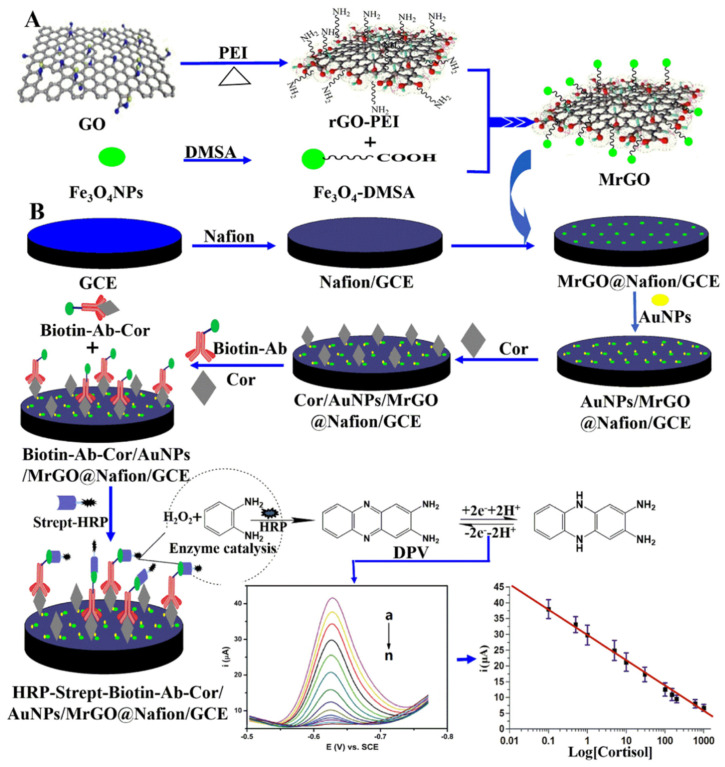
Scheme of the construction of the electrochemical cortisol immunosensor; (**A**) GO functionalization; (**B**) surface functionalization and detection mechanism (reproduced with permission from [67]. Copyright 2017 Elsevier).

Sun and co-workers developed a sensitive and competitive electrochemical immunosensor for cortisol detection [67]. This immunosensor consists of gold nanoparticles and magnetically functionalized reduced graphene oxide (AuNPs/MrGO) (Figure 10). Cortisol is detected by the presence of a cortisol-selective antibody on the surface. The final signal of the electrochemical immunosensor in the test fluid had negative correlations with the cortisol concentration in the samples. Finally, the electrochemical response of the immunosensor was greatly amplified by applying AuNPs/MrGO with excellent electrical conductivity. The cortisol detection ability was excellent, with a linear range between 0.1 to 1000 ng/mL, and a detection limit of 0.05 ng/mL. This system has been tested with human serum as the real sample, and thus a device for obtaining saliva or sweat should be implemented for the space mission.

**Figure 11:**
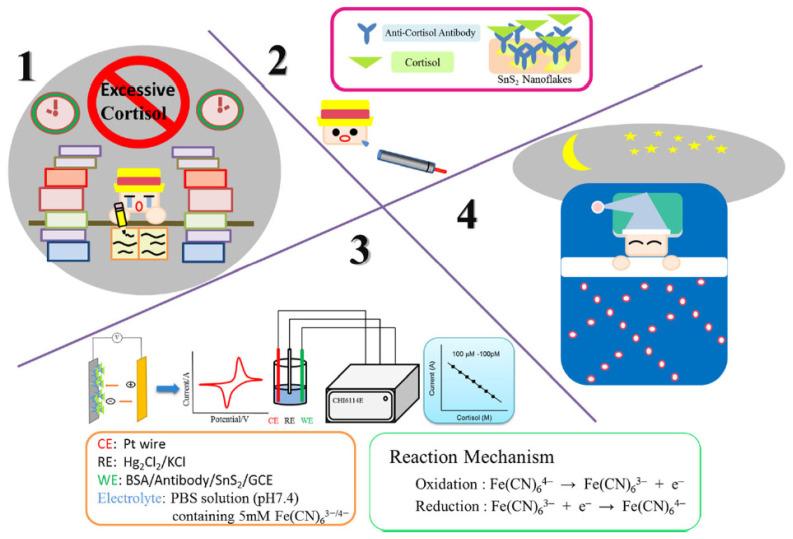
The schematization of the new type of rapid point-of-care test for the detection of salivary cortisol (reproduced with permission from [68]. Copyright 2019 Springer).

Liu and co-workers reported on a new type of rapid point-of-care test for the non-invasive detection of salivary cortisol [68] (Figure 11). The sensor was prepared by loading onto glassy carbon electrodes (GCE) multilayer films containing two-dimensional tin disulfide nanoparticles [69], cortisol antibody (C-M_ab_), and bovine serum albumin (BSA). The electrochemical performance of the biosensor as a function of cortisol concentrations was determined by cyclic voltammetry and differential pulse voltammetry, showing a good detection range from 100 pM to 100 μM in authentic saliva samples, a detection limit of 100 pM, and a sensitivity of 0.0103 mA/Mcm^2^ (R^2^ = 0.9979). The detection of cortisol in real saliva requires only 2 mL of sample, thus making it usable in practical analysis.

**Figure 12:**
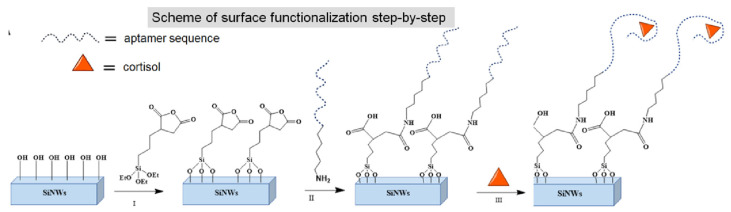
Schematic diagram of the surface functionalization of SiNW with the cortisol-specific aptamer (adapted with permission from [70]. Copyright 2020 American Chemical Society).

Klinghammer and co-workers designed a portable microfluidic platform based on silicon-based semiconductor nanowire sensors (SiNW FETs) for monitoring diurnal cortisol levels in saliva samples at nanomolar concentrations [70]. The sensor is based on nanoscopic field-effect transistors (FETs), which provide real-time and label-free detection due to their low detection limits [71]. To bring the ‘target-receptor’ complex closer to the surface of the nanowires (SiNWs), they exploited the specific DNA aptamer sequences previously reported by Martin et al. [72]. In addition, aptamer-based FETs are suitable sensors for the detection of small analytes like cortisol. The sensing mechanism is based on the conformational changes induced by cortisol upon binding to negatively charged aptamers. Cortisol wraps tightly around silicon nanowires, and the surface potential, which is measured by static contact angle measurements, is altered (Figure 12). Finally, circular dichroism (CD) spectroscopy was used to test the selectivity of the platform and the conformational changes observed following the recognition of cortisol levels. As previously cited, microfluidic devices cannot be used in the absence of gravity.

**Figure 13:**
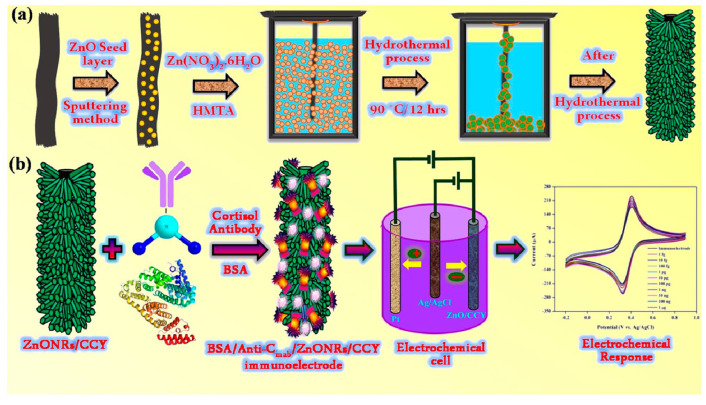
Schematic illustration of (**a**) insertion of ZnONRs on CCY and (**b**) step-by-step realization of an immunosensor with ZnONRs/CCY and electrochemical detection of cortisol (reproduced with permission from [73]. Copyright 2020 American Chemical Society).

Madhu and co-workers designed highly flexible, wettable yarn-based electrochemical immunosensors capable of non-invasively quantifying levels of biochemical markers on human sweat in real-time [73]. Specifically, these yarn-based sensors have been immobilized via hydrothermal synthesis on ZnO nanorods (ZnONRs) as they exhibit excellent morphology, crystallinity, and specific surface area. The ZnONRs showed excellent mechanical stability and super-wetting properties (Figure 13). With this immunosensor, cortisol could be detected over a wide linear detection range from 1 fg/mL to 1 μg/mL. The detection limits were calculated between 0.45 and 0.098 fg/mL using the CV and DPV techniques, respectively. Cortisol was detected in real sweat samples, making this system interesting for practical applications if it can be transformed into a portable device.

**Figure 14:**
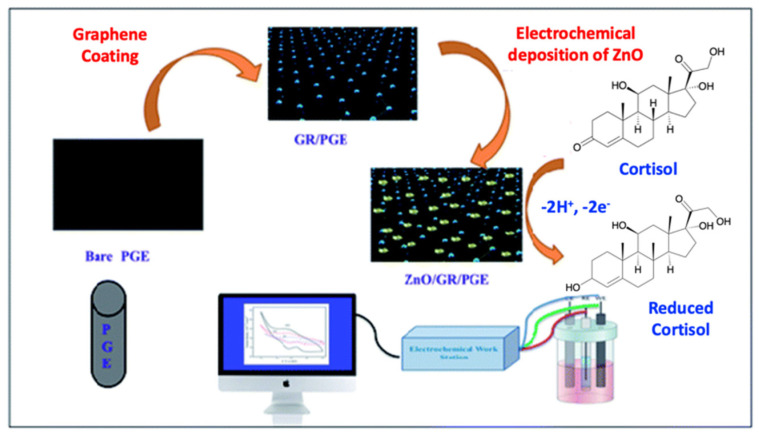
Schematic of the electrocatalytic determination of cortisol on ZnO nanoparticles deposited on a pencil graphite electrode (PGE) (adapted with permission from [74]. Copyright 2021 Royal Society of Chemistry).

Rison and co-workers developed a new electrochemically conductive sensing electrode, based on ZnO nanoparticles electrochemically deposited on a pencil graphite electrode (PGE) coated with graphene [74] (Figure 14). The sensor was characterized using electrochemical techniques such as CV analysis and electrochemical spectroscopic impedance testing (EIS). This system can detect salivary cortisol amounts within a linear range of 5 × 10^−10^ M–115 × 10^−10^ M, with a LOD of 0.15 nM. These limits, the LOD, the LOQ, and the linearity interval were measured by Differential Pulse Voltammetry (DPV) studies. The possibility to detect cortisol with CV and EIS makes this system suitable for laboratory scales, and thus the practicability for real analysis should be improved.

**Figure 15:**
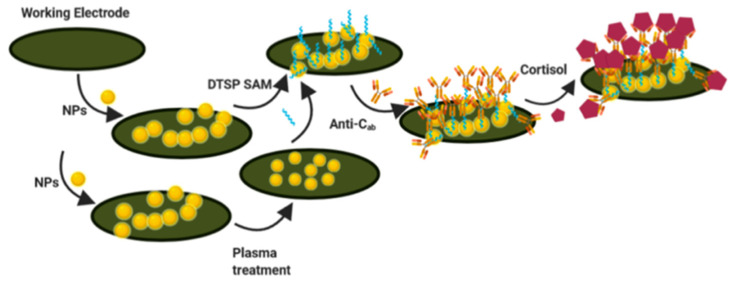
Steps in the fabrication of a cortisol immunosensor on untreated (drop-cast) and AuNP-modified plasma-treated electrodes (reproduced with permission from [75]. Copyright 2021 American Chemical Society).

Sonawane and co-workers demonstrated the improved distribution and electronic properties of gold nanoparticles (AuNPs) using a plasma-assisted technique at room temperature [75] (Figure 15). This sensor was used for the electrochemical detection of cortisol by exploiting the plasma-induced effects of AuNPs. The electrochemical immunosensor was realized using AuNPs by depositing them onto the surface of the activated screen-printed carbon electrode (SPCE). AuNPs were modified with dithiobis-(succinimidyl propionate) (DTSP) to form SAMs and conjugate anti-cortisol antibodies. The electrochemical sensing of cortisol occurs through antigen–antibody interactions on the surface of the modified electrode. To demonstrate the improvement of the electronic properties of the AuNPs after the plasma-assisted approach, surface-enhanced Raman spectroscopy (SERS) was used via ζ-potential measurements. Using electrochemical techniques such as cyclic voltammetry and electrochemical impedance spectroscopy, the analytical characteristics of the cortisol sensor were studied. Additionally, this system works on a laboratory scale and needs to be implemented to make it portable.

**Figure 16:**
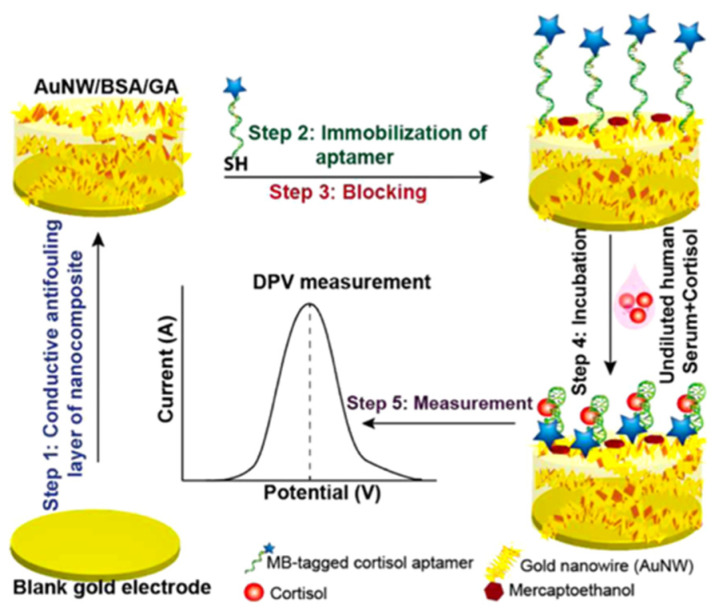
Fabrication of the aptasensor. (Step 1) Synthesis of a nanocomposite layer on a gold electrode (BSA/AuNW/GA). (Step 2) Immobilization of the thiolated and MB-labeled cortisol aptamer, followed by (Step 3) Blocking with mercaptoethanol. (Step 4) Incubation with the sample and (Step 5) DPV measurement (reproduced with permission from [76]. Copyright 2021 American Chemical Society).

Singh and co-workers developed a reagent-free electrochemical aptasensor with a nanocomposite antifouling layer to monitor in vivo stress biomarkers such as cortisol [76]. This electrochemical sensor is based on a conformation-switching aptamer, on top of a conductive antifouling nanocomposite surface, to detect cortisol in undiluted human serum, as shown in Figure 16. In particular, it consists of a thiolated aptamer, labeled with methylene blue (MB), immobilized on a gold nanowire nanocomposite (AuNW) to capture cortisol and generate a signal proportional to the cortisol concentration. The signal is recorded by differential pulse voltammetry (DPV) and chronoamperometry. The aptasensor shows excellent stability in undiluted human serum and sensitive response with detection limits of 0.51 and 0.68 nM in buffer and undiluted serum samples, respectively. A linear detection range of 1 to 1000 nM was calculated for artificial samples in both buffer and serum, which covers the physiological cortisol range of 100–600 nM in humans. This sensor paves the way for the realization of new biosensor formats, such as implantable and wearable sensors, by achieving performance comparable to the best cortisol test. The use of human serum as a real sample limits the application in space missions and, in general, in facile cortisol detection.

Madhu and co-workers designed new wearable electrochemical cortisol-sensing devices, exploiting the anchoring of nano-structured materials on textile materials [77]. The sensor exploits a conductive carbon fiber integrated with SnO_2_ nanoflakes (SnO_2_/CCY). Anti-cortisol antibodies were immobilized on this fiber to improve its detection selectivity. Using an electroactive redox probe (Fe(CN)_6_^3−/4−^), the response of the electrochemical immunosensor to cortisol binding was monitored, and the oxidation current of the redox probe (−0.42 V vs. Ag/AgCl) was probed to create the calibration plot of the sensor. Finally, the electrochemical immunosensor responded to a wide range of cortisol concentrations (from 10 fg/mL to 1 μg/mL) and showed a good detection limit (1.6 fg/mL). Under optimized conditions, the immunosensor was evaluated to measure the cortisol levels in human sweat at a laboratory scale. Due to the good analytical parameters, such as linearity and detection limit, the development of a practical device will lead to a good system for real cortisol detection.

## 5. Conclusions and Future Perspectives

This review summarizes recent scientific development on miniaturized sensor devices, based on gold and carbon nanoparticles, nanotubes, nanowires, and nanoelectrodes, which enable the rapid, real-time analysis of cortisol levels in biological samples (such as saliva, urine, sweat, and plasma) to monitor human health conditions under extreme stress. These nanomaterials will be used as probes within portable point-of-care or lab-on-chip smart sensing devices, able to quantitatively monitor cortisol levels through a quick and easy analytical protocol, which is of huge importance for space applications, as cortisol levels vary with daily activities. In particular, we have focused on the optical and electrochemical detection of cortisol using nanostructures, due to their high sensitivity. In order to obtain a promising sensoristic device for the continuous monitoring of cortisol in space, we believe that the combination of a specific recognition site (aptamers or antibodies) with nanoparticles (as a transducer), exploiting an optical/colorimetric readout can be the ideal setup. In particular, nanoparticles (carbon or gold) can be easily functionalized on their surface by stable bonds with cortisol-selective aptamers or antibodies. The possibility to monitor the detection of the analyte by a simple change of color/emission by using a smartphone or optical fiber as a detector makes this ideal sensor device portable and suitable for real applications. Due to the presence of cortisol in different human matrices, sensing can be performed in blood, urine, saliva, sweat, hair, and interstitial fluid. The sampling of urine, sweat, and saliva is undoubtedly the most convenient and least invasive sampling technique; however, the low cortisol concentration values require high sensitivity and selectivity. These two aspects will be the crucial target for future cortisol detection by point-of-care techniques. The sensoristic devices here reported are, until now, developed on Earth, and are required to be implemented to be used in a real space mission. To this end, some important aspects need to be addressed: (*i*) the absence of gravity, (*ii*) portability, (*iii*) wearability, (*iv*) the use of non-dangerous materials (inflammable materials should be avoided), (*v*) possibility to restore the sensoristic device, to reduce the hindrance on board, (*vi*) easy to use by the crew members. The development of efficient point-of-care sensors for cortisol detection is important also for stress diagnosis, and in general for the monitoring of cortisol, on the Earth, giving the possibility of an easy analysis methodology to be used by common persons.

## Figures and Tables

**Table 1:** Cortisol levels in different biological samples in nominally healthy subjects. Adapted with permission from [17]. Copyright 2020 Elsevier.

Sample	Cortisol Concentrations
Blood	25 mg/mL (morning), 2 mg/mL (midnight)
Urine ^a^	21,458–149,696 ng/24 h 44,000–140,000 ng/24 h
Saliva	1–12 ng/mL (morning), 0.1–3 ng/mL (evening)
Sweat	8–142 ng/mL
Hair	18–153 pg/mg
Interstitial fluid (ISF)	12–34 ng/mL (morning), 9–13 ng/mL (evening)

^a^ The level of cortisol in the urine is measured over a 24 h period and is referred to as the 24 h urinary free cortisol test; this test consists of the collection of a subject’s urine for 24 h; consequently, the results are expressed in 24 h units due to the difference in the amount that can be collected from subject to subject. This test measures the total amount of free cortisol excreted by the subject over 24 h, regardless of the amount of urine.

## Data Availability

Not applicable.
